# *Zanthoxylum alatum* abrogates lipopolysaccharide-induced depression-like behaviours in mice by modulating neuroinflammation and monoamine neurotransmitters in the hippocampus

**DOI:** 10.1080/13880209.2017.1391298

**Published:** 2018-03-23

**Authors:** Chandana Choudhury Barua, Prakash Haloi, Beenita Saikia, Kunjbihari Sulakhiya, Debesh Chandra Pathak, Shantanu Tamuli, Hooriah Rizavi, Xinguo Ren

**Affiliations:** a Department of Pharmacology and Toxicology, College of Veterinary Science, Assam Agricultural University, Guwahati, India;; b National Institute of Science Education and Research Bhubaneswar (HBNI), School of Biological Sciences, Khurdha, India;; c Department of Pharmacy, Indira Gandhi National Tribal University (IGNTU), Amarkantak, India;; d Department of Pathology, College of Veterinary Science, Guwahati, India;; e Department of Animal Biochemistry, College of Veterinary Science, Assam Agricultural University, Guwahati, India;; f Department of Psychiatry, Molecular Biology Research Building (MBRB), University of Illinois, Chicago, IL, USA

**Keywords:** Cytokines, dopamine, imipramine, norepinephrine, serotonin

## Abstract

**Context:** Depression is an inflammatory, commonly occurring and lethal psychiatric disorder having high lifetime prevalence. *Zanthoxylum alatum* Roxb. (Rutaceae), commonly called Timur, has high medicinal value and is used ethnomedicinally for the treatment of various diseases.

**Objective:** To evaluate the effect of hexane extract of *Z. alatum* seeds (ZAHE) on lipopolysaccharide (LPS)-induced depression-like behaviour in Swiss albino mice.

**Materials and methods:** Mice were treated with ZAHE (100 and 200 mg/kg, p.o.) and imipramine (10 mg/kg injected i.p.) for 14 days. On 14th day of the treatment, depression-like behaviour was induced by LPS (0.83 mg/kg injected i.p.) and after 24 h of LPS administration, it was assessed by measuring behavioural parameters and biochemical estimations.

**Results:** Behavioural tests, including the open field test, forced swimming test, tail suspension test and sucrose preference test revealed that ZAHE (100 and 200 mg/kg, p.o.) and imipramine (10 mg/kg injected i.p.) alleviated the depression symptoms of LPS-induced mice. Moreover, ZAHE treatments reversed the LPS-induced alterations in the concentrations of norepinephrine and serotonin (5-HT) and inhibited the expression of brain-derived neurotrophic factor, pro-inflammatory cytokines and oxido-nitrosative stress in the mice. Acute toxicity was calculated to be LD_50_ > 2500 mg/kg.

**Discussion and conclusions:** This study showed that LPS-induced depression in mice was significantly prevented by ZAHE at both the dosages. In conclusion, ZAHE exhibited an antidepressant activity by altering monoaminergic neurotransmitters in the brain combined with its anti-inflammatory potential. Thus, it could be an effective therapeutic against inflammation-induced depression and other brain disorders.

## Introduction

Major depressive disorder (MDD) is a mental disorder characterized by low mood, loss of interest or pleasure in normally enjoyable activities, fluctuation in body weight (increase or decrease), and anorexia (Martin-Soelch 2009). It occurs mostly in people between the ages of 30 and 40 with 8–12% prevalence rate (Eaton et al. 1997; Andrade et al. 2003; Kessler et al. 2003).

Research has shown a possible relationship between inflammation and pathogenesis of MDD (Maletic and Raison 2009; Miller et al. 2009). Stressful life events, such as sickness, may prompt a physical response that actually alters the brain function. The administration of the bacterial endotoxin lipopolysaccharide (LPS) induces sickness behaviour in animals, which resembles depression-like symptoms, including anhedonia, anorexia and weight loss (Yirmiya 1996; De La Garza 2005; Singal et al. 2006; Henry et al. 2008).

*Zanthoxylum alatum* Roxb. (Rutaceae) is an evergreen small xerophytic medicinal tree or shrub, native to Himalayan regions in India. It is commonly known as Indian Prickly Ash, Nepal pepper and toothache tree but locally known as Tejphal (Hindi), Tejowati (Sanskrit), Mukthrubi (Manipur), Timur (Nepal). The ethnomedicinal importance of bark, fruits and seeds of *Z. alatum* (ZA) have been known since antiquity in indigenous system of medicine as carminative, stomachic and anthelmintic (Singh and Singh 2011). The fruit and seeds are also used as an aromatic tonic in fever and dyspepsia. In Nepal folk medicine, *Z. alatum* is used in cold and cough, tonsillitis, headache, fever, vertigo, diarrhoea and dysentery (Geweli and Awale 2008). In Indian folk medicine, it is used in the treatment of fever, dyspepsia, and cholera (Chopra et al. 1986). Moreover, powdered fruit mixed with *Mentha* species and table salt is given with boiled egg for the chest infection and digestive problems (Islam et al. 2009).

*Zanthoxylum alatum* possesses larvicidal (Tiwary et al. 2007), hepatoprotective, antioxidant (Ranawat et al. 2010), anti-nociceptive, anti-inflammatory and antipyretic activities (Guo et al. 2011) also. It contains various phytopharmaceuticals, such as berberine, dictamnine, xanthoplanine, armatamid, asarinin, fargesin, α- and β-amyrins and lupeol (Kalia et al. 1999; Nadkarni 2002). The present study evaluates the effect of seeds of *Z. alatum* extract in LPS-induced depression-like behaviour and concurrent biochemical changes in mice.

## Materials and methods

### Chemicals

LPS from *Escherichia coli,* strain 055:B5, serotonin hydrochloride, dopamine hydrochloride, (±)-norepinephrine (NE) (+)-bitartrate salt, imipramine hydrochloride were procured from Sigma-Aldrich Corp., St. Louis, MO. All other chemicals used were of analytical grade.

### Animals

Male Swiss albino mice (weighing 18–22 g) were obtained from the animal facility of the Department of Pharmacology and Toxicology, College of Veterinary Science, Khanapara, Assam, India. They were housed in polypropylene cages and acclimatized for a week under standard conditions (temperature – 22 ± 3 °C, humidity – 50 ± 10%, and 12:12 h light/dark cycle). The experimental mice were given free access to standard pellet diet and water *ad libitum*. All experimental protocols and methods were approved by Institutional Animal Ethical Committee (IAEC) of College of Veterinary Sciences, Assam Agricultural University (770/ac/CPCSEA/FVSc, AAU/IAEC/15-16/367). Laboratory animal handling and experimental procedures were performed in accordance with the guidelines recommended by Committee for the Purpose of Control and Supervision of Experiments on Animals (CPCSEA), Ministry of Environment, Forests, and Climate Change, Government of India.

### Plant material and preparation of extract

*Zanthoxylum alatum* seeds were collected in the month of July 2015 from Arunachal Pradesh, India. The seeds were authenticated by Dr. I. C. Barua, Principal Scientist, Department of Agronomy, Assam Agricultural University and a voucher specimen (5109 dated 25 September 2014) was deposited in the Herbarium. The seeds were cleaned and air dried for a week at 35–40 °C and was pulverized in an electric grinder. Preparation of hexane extract of *Z. alatum* (ZAHE) was done as per the standard methods using Soxhlet extraction apparatus (3840-Extraction Apparatus, Borosil Glass Works Limited, Mumbai, India) until the solvent extracted showed no more colour. The extract was concentrated under reduced pressure using Rotary Evaporator (BUCHI, ROTAVAPOR, R-210; Switzerland) to yield the viscous crude extract. The recovery percentage of ZAHE with respect to dry powder was found to be 8.36% w/w.

### Experimental design

The mice were randomly divided into five groups (*n* = 6 mice/group).

Group 1: Vehicle control and receive Tween 80 and saline.

Group 2: Negative control and receive saline and LPS (0.83 mg/kg BW, i.p.).

Group 3: Standard or positive control and receive imipramine (10 mg/kg BW, i.p.) and LPS (0.83 mg/kg BW, i.p.).

Group 4: Receive ZAHE (100 mg/kg BW) and LPS (0.83 mg/kg BW, i.p.).

Group 5: Receive ZAHE (200 mg/kg BW) and LPS (0.83 mg/kg BW, i.p.).

The vehicle control, LPS and ZAHE treated groups were given respective treatment once daily for 14 consecutive days. For the pretreatment paradigm, imipramine (10 mg/kg) was administered intraperitoneally to the animals for 14 days (Mello et al. 2013). On the last day of the treatment, i.e., 14th day, LPS (0.83 mg/kg) was injected intraperitoneally after 30 min of respective treatment to all groups except vehicle control group. After 24 h of LPS administration, battery of behavioural tests including open field test (OFT), forced swimming test (FST), tail suspension test (TST) and sucrose preference test (SPT) were performed to evaluate the depressive-like behaviour in mice (Sulakhiya et al. 2014). Following the behavioural studies, mice were sacrificed and brains were quickly dissected out to isolate hippocampus (HC). All the tissues were stored at –80 °C until further analysis.

### Acute toxicity study

Acute oral toxicity study was performed as per Organization for Economic Co-operation and Development 423 guideline. The overnight fasted mice (*n* = 3) were orally administered ZAHE at the limit dose of 2000 mg/kg and observed continuously for behavioural, neurological and autonomic profiles for 2 h and then 24 h, 72 h and thereafter up to 14 days for any lethality, moribund state or death. The limit test was repeated in another group of mice (*n* = 3) for confirmation and toxic class of LD_50_ determination. Acute oral toxicity study showed no toxicity or moribund stage due to ZAHE treatment. This suggested that non-observable adverse effect dose level is more than 2000 mg/kg and approximate LD_50_ is greater than 2500 mg/kg.

### Behavioural assessments

#### Open field test

To assess the possible effects of the ZAHE on locomotor activity, mice were subjected to the open-field test. The ambulatory behaviour was assessed in an open-field test with minor modifications (Rodrigues et al. 1996, 2002). They were individually placed into a clean, novel cage similar to the home cage, but devoid of bedding or litter. The cage was divided into 12 virtual quadrants, and locomotor activity was measured by counting the number of line crossings and rearing during 5 min period. The apparatus was then cleaned with a 10% alcohol solution and dried after each mouse tested to prevent olfactory clue of the previous animal.

#### Forced swimming test

FST was performed to assess the despair behaviour of the rodents (Porsolt et al. 1977). The test was performed for mice using Porsolt Forced Swimming test apparatus (Stoelting Co., Wood Dale, IL). Water temperature was maintained at 30 ± 1 °C. Mice were placed in an inescapable cylinder for 6 min during the test session and video recorded using ANY-maze software (Stoelting Co., USA). Immobility time was counted for the last 4 min. They were considered immobile when ceased struggling, remained floating motionless and only made those movements necessary to keep their head above the water.

#### Tail suspension test

TST was conducted using tail suspension test apparatus (Stoelting Co., USA) as described by Steru et al. (1985). Mice were individually suspended in the hook of the TST box, 60 cm above the surface of a table with an adhesive tape placed 1 cm away from the tip of the tail in a dark room. The immobility time of each mouse was video recorded using ANY-maze software (Stoelting Co., USA) for 6 min and immobility time was counted for the last 4 min of the total 6 min observation period.

#### Sucrose preference test

The sucrose preference test was employed to evaluate anhedonia (response to reward) (Henry et al. 2008). Before testing, all mice were acclimatized to drinking water and 2% sucrose solution for 5 days before LPS administration to establish a baseline sucrose preference for each mouse. Sucrose solution was filled in drinking bottle having stopper valve and placed in the home cage of animals. The relative position of bottles was changed daily to avoid development of a place preference. On the day of testing, mice were deprived of fluid and food for 2 h prior to testing. At the end of the testing, i.e., 48 h post-LPS administration, fluid content was measured and sucrose preference was calculated using the following equation: Sucrose preference (%) = sucrose intake/(sucrose intake + water intake) × 100.

#### Food consumption and body weight

Food intake and body weight were recorded once daily at the onset of the dark period. Food containers were filled with 50 g of the pelleted mice chow, and food intake was quantified 2 and 24 h after LPS/saline injection. Consumption of food in grams were recorded by subtracting the food remaining in the food container and on the cage floor from the amount of food measured at the preceding time point. Food spillage in the cage was ignored because it has been previously reported to be similar among rats/mice and generally weigh less than 1% of the food consumed (Yirmiya 1996). Body weight was also taken at 2 and 24 h after LPS/saline injection. Both food intake and body weight were expressed in grams (g).

### Assessment of oxidative stress and antioxidant status

#### Lipid peroxidation (LPO) and nitric oxide (NO)

The LPO end product malondialdehyde (MDA) was estimated in the HC by Ohkawa et al. (1979) method using the thiobarbituric acid and the optical density was measured spectrophotometrically at 532 nm. The values are expressed as ηM of MDA/mg of protein. Nitrite, an indicator of the production of NO was determined by a colourimetric assay using Griess reagent (Sigma-Aldrich, St Louis, MO). The concentration of nitrite was determined from a sodium nitrite standard curve and expressed as μM of nitrite/mg of protein (Miranda et al. 2001).

#### Antioxidant status

Reduced glutathione (GSH) was estimated according to the method described by Ellman (1959). The concentration of reduced GSH was expressed as µM of GSH/mg protein. Superoxide dismutase (SOD) activity was estimated using SOD assay kit (Sigma-Aldrich, St Louis, MO) according to the manufacturer’s instructions. The SOD activity (units/mg of protein) was calculated by using the standard plot. The Catalase (CAT) activity was determined according to the method of Sinha (1972). CAT activity was expressed as µM of H_2_O_2_ decomposed/min/mg protein. GSH peroxidase (GPx) was estimated as described by Rotruck et al. (1973). The total protein was estimated by the method of Bradford (1976).

### Neurochemical determinations

#### Estimation of brain cytokines and brain-derived neurotrophic factor (BDNF)

Mice HC samples were homogenized in 0.9% normal saline (w:v 1:9) and then centrifuged (4 °C) at 3500 rpm for 10 min. Dilutions of protein standards and samples were added to 96-well ELISA plates, followed by the addition of a biotin-conjugated anti-cytokine antibody and avidin–horseradish peroxidase complex according to the manufacturer’s instructions. A colour reaction was achieved with the addition of a substrate solution and was terminated using the stopping solution. The concentrations of pro-inflammatory cytokines (TNF-α, IL-1β, and IL-6), anti-inflammatory cytokines (IL-10), and BDNF were quantified according to the optical density, as detected using a microplate reader (450 nm) (MultiScan Go, Thermo Fisher Scientific Oy. Ratastie 2, Vantaa, Finland). A standard curve was made, and the experimental results (shown as picograms per millilitre) were analyzed from within the curve (Xue et al. 2015).

#### Estimation of catecholamines

Levels of NA, DA, and 5-HT were estimated using high-performance liquid chromatography (UHPLC) (Thermo Scientific, Dionex Ultimate 3000 model and Chromeleon 7 software, Waltham, MA) coupled with an electrochemical detector (ESA Coulochem^®^ III detector, ESA Biosciences, Inc. Chelmsford, USA) as described by Kim et al. (1987). Typically, a 0.3-N perchloric acid (PCA) solution is added to the tissue sample for preservation and extraction of catecholamines and acid metabolites. The tissue sample was weighed and then placed into a microcentrifuge tube. For every 100 µg of wet weight, a volume of 1.0 mL of 0.3 N PCA is added. Then the samples were pulse sonicated using a sonicator (LABSONIC^®^ P Sartorius Stedim Biotech, Göttingen, Germany) on ice in this solution to degrade any native enzyme activity and help precipitate the proteins from the sample. The samples were then centrifuged for 10–15 min (time depends on *g*-force of the centrifuge) to form a pellet and clear supernatant that is free of particulates. An aliquot of the supernatant was finally diluted 1:2 with readymade HPLC mobile phase. HPLC chromatographic conditions using a normal bore column approach was: flow: Isocratic at 0.60 mL/min; temperature: 32 °C; column: MD-150 column, guard column and holder; injection volume: 10–20 µL partial loop; mobile phase: 75 mM NaH_2_PO_4_, 1.7 mM 1-octanesulfonic acid, 100 µL/litre triethylamine, 25 mM EDTA, 10% acetonitrile adjusted to pH 3 with phosphoric acid; Coulochem detector: 5011 A cell:E1 at –150 mV: E2 at +220 mV, 5020 cell: EGC at +250 mV.

### Statistical analysis

Statistical analysis was performed using Graph Pad Prism (version 5.0, Graph Pad Software Inc., San Diego, CA) Software. All data were expressed as mean ± standard error of the mean (SEM). The data were statistically analyzed using one-way analysis of variance followed by Dunnett’s *post hoc* test. Results were considered statistically significant when *p* > 0.05.

## Results

### Acute toxicity study

Acute oral toxicity studies revealed no lethality or any toxic reactions or moribund state up to the end of the study period. ZAHE was safe up to a dose level of 2000 mg/kg of body weight (limit test) and the observed LD_50_ for oral administration of the extracts was more than 2500 mg/kg. Hence the two doses, namely, 100 and 200 mg/kg body weight used in the study were also free from toxic effects.

### Behavioural assessment

#### Effect of ZAHE on line crossings and rearing tested in OFT

**Figure 1.**
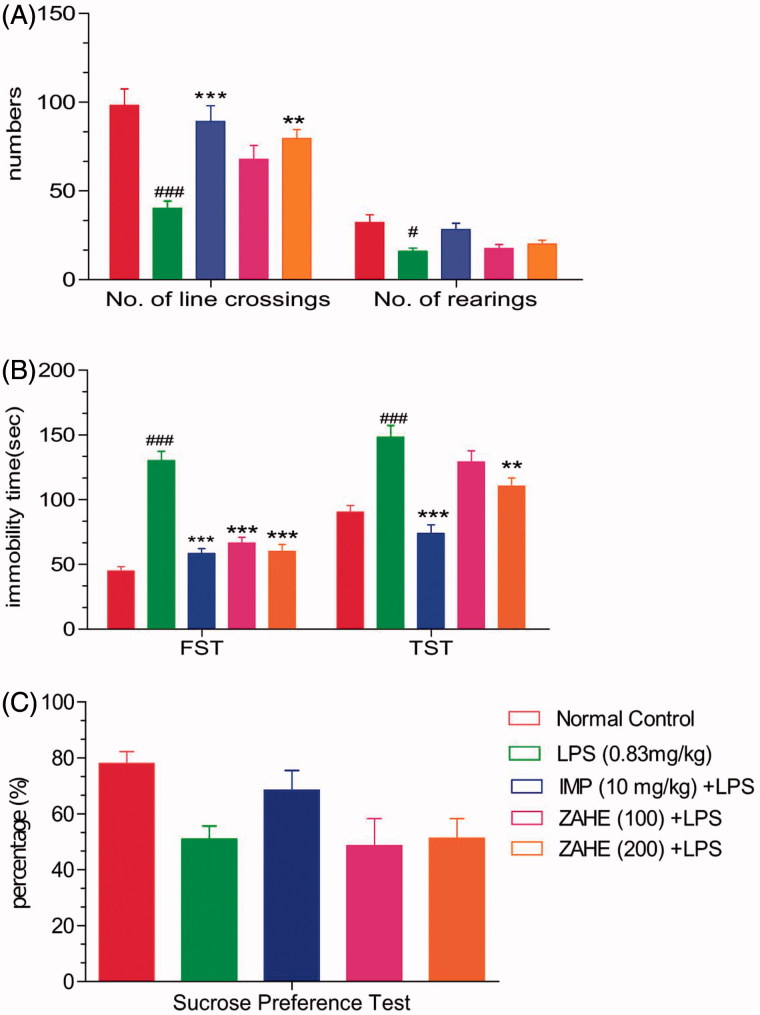
Effect of ZAHE pretreatment in LPS challenged animals on behavioural parameters: (A) Open field test, (B) FST and TST, and (C) sucrose preference test. Values represent the mean ± SEM. (*N* = 6 animals/group). ###*p* < 0.001, ##*p* < 0.01, #*p* < 0.05 compared with normal control. *p < 0.05; **p < 0.01; ***p < 0.001 compared with LPS-challenged group.

As indicated in Figure 1(A), LPS and ZAHE produced significant difference in number of crossings and rearing as compared to vehicle-treated group. Statistically significant (*p* > 0.05) decline in movement (crossing) and rearing in case of LPS-treated mice was noticeable.

#### Effect of ZAHE on immobility time tested in FST

In the FST, the results predicted that LPS challenged mice showed significant (*p* > 0.001) increase in immobility duration as compared to vehicle-treated group. Pretreatment with ZAHE reversed LPS-induced increase in immobility duration at both the dosages, i.e., 100 and 200 mg/kg (*p* > 0.001). Mice treated with imipramine showed significant (*p* > 0.001) reduction in immobility time compared to LPS-treated group (Figure 1(B)).

#### Effect of ZAHE on immobility time tested in TST

The LPS-treated group showed significant (*p* > 0.001) increase in the duration of immobility as compared with that of the vehicle-treated group, whereas, pretreatment with ZAHE (100 and 200 mg/kg) significantly (*p* > 0.01) reversed LPS-induced increase in immobility duration (Figure 1(B)). On the other hand, the standard drug, i.e., imipramine (10 mg/kg, i.p.) showed significant (*p* > 0.001) decrease in immobility duration as compared to LPS-challenged animals.

#### Effect of ZAHE on anhedonic behaviour tested in SPT

LPS-challenged mice produced marked anhedonia, which is evident from reduction in the sucrose preference in LPS-treated group as compared with vehicle-treated group. Moreover, ZAHE (100 and 200 mg/kg) pretreatment significantly prevented anhedonia (Figure 1(C)).

#### Effect of ZAHE on food consumption and changes in body weight

**Figure 2.**
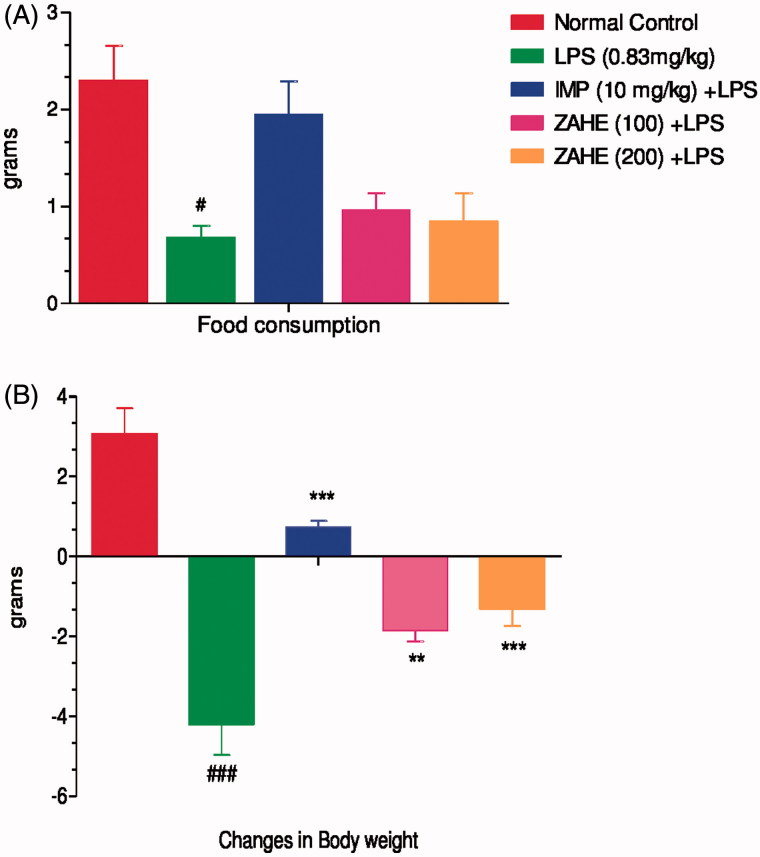
Effect of ZAHE pretreatment in LPS challenged animals on: (A) changes in body weight and (B) food consumption after 24 h LPS administration on day 15. Values represent the mean ± SEM. (*N* = 6 animals/group). ###*p* < 0.001, ##*p* < 0.01, #*p* < 0.05 compared with normal control. *p < 0.05; **p < 0.01; ***p < 0.001 compared with LPS-challenged group.

LPS challenge led to a marked decrease in food consumption and changes body weight in mice. The reduction in both food intake and changes in body weight were significantly (*p* < 0.05) improved by imipramine and chronic pretreatment of ZAHE at both the dosages (100 and 200 mg/kg) (Figure 2(A,B)).

### Biochemical estimation

#### Effect of ZAHE on in vivo oxidative stress and antioxidant markers

**Figure 3.**
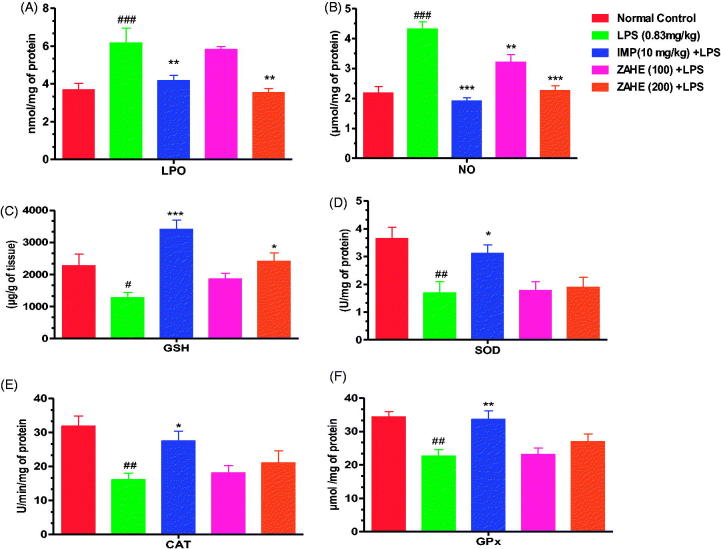
Effect of ZAHE pretreatment in LPS challenged animals on *in vivo* oxidant and antioxidant parameters: (A) LPO, (B) nitrite level, (C) reduced GSH, (D) SOD, (E) catalase, and (F) GPx. Values represent the mean ± SEM. (*N* = 6 animals/group). ###*p* < 0.001, ##*p* < 0.01, #*p* < 0.05 compared with normal control. *p < 0.05; **p < 0.01; ***p < 0.001 compared with LPS challenged group.

Figure 3(A) showed that LPS after 24 h enhanced the thiobarbituric acid reactive substances (TBARS) level significantly in the HC (*p* > 0.001) when compared with vehicle-treated group. Imipramine (10 mg/kg) and ZAHE (200 mg/kg) pretreatment resulted in significant reduction (*p* > 0.01) in TBARS content in the HC. Moreover, ZAHE (100 mg/kg) pretreatment also showed beneficial effect on TBARS content in the HC which was not significant. Figure 3(B) showed, nitrite level was elevated significantly in the HC (*p* > 0.001) of mice after 24 h of LPS administration as compared to that of vehicle-treated group. Imipramine (10 mg/kg) and ZAHE (200 mg/kg) pretreatment prevented the LPS-induced increase in nitrite level in the HC significantly (*p >* 0.001) (Figure 3(B)). In addition, ZAHE at dose of 100 mg/kg also showed significant (*p >* 0.05) protective effect against LPS-induced decrease in hippocampal nitrite level in mice. Figure 3(C) showed that ZAHE (200 mg/kg) pretreatment significantly (*p* > 0.05) elevated GSH level when compared with LPS-treated group, but 100 mg/kg pretreatment was ineffective. However, imipramine improved hippocampal GSH level significantly (*p >* 0.001) as compared to LPS-treated mice. As depicted in Figure 3(D), LPS evoked significant decrease in SOD (a class of enzymes that catalyzes the reduction of superoxide to hydrogen peroxide) activity which was improved by imipramine pretreatment significantly (*p >* 0.05) but both the dosages of ZAHE (100 and 200 mg/kg) did not show any significant effect against LPS-induced decrease in hippocampal SOD. Also, LPS challenged mice presented a decreased CAT (a relevant endogenous antioxidant enzyme responsible for hydrogen peroxide detoxification) activity, which was elevated following treatment with imipramine (*p >* 0.05) but ZAHE pretreatment at both the dosages did not show any significant effect (Figure 3(E)). Furthermore, LPS significantly (*p >* 0.01) decreased GPx (important endogenous antioxidant enzymes) content in the HC in LPS challenged mice which was improved by imipramine pretreatment in significant manner (*p >* 0.01) but ZAHE was ineffective at both the dosages (Figure 3(F)).

#### Effect of ZAHE on hippocampal cytokines and BDNF level

**Figure 4.**
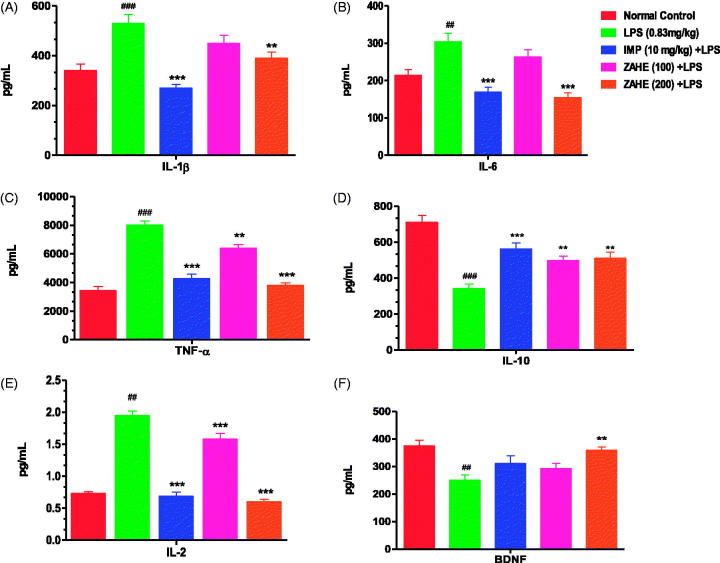
Effect of ZAHE pretreatment in LPS challenged animals on hippocampal cytokines and BDNF level: (A) IL-1β, (B) IL-6, (C) TNF-α (D) IL-10, (E) IL-2, and (F) BDNF. Values represent the mean ± SEM. (*N* = 6 animals/group). ###*p* < 0.001, ##*p* < 0.01, #*p* < 0.05 compared with normal control. *p < 0.05; **p < 0.01; ***p < 0.001 compared with LPS-challenged group.

The proinflammatory cytokine, IL-1β level was significantly (*p* > 0.001) increased after 24 h of LPS administration in HC of mice which was significantly prevented by ZAHE (200 mg/kg) pretreatment (*p* > 0.01), whereas, ZAHE (100 mg/kg) pretreatment was able to avert IL-1β level in HC of LPS challenged mice but not in significant manner (Figure 4(A)). The standard drug, i.e., imipramine significantly (*p >* 0.001) thwarted the increase in the hippocampal IL-1β level induced by LPS (Figure 4(A)). The IL-6 level was also significantly increased in LPS challenged mice (*p* > 0.01). Imipramine and higher dose (200 mg/kg) of ZAHE significantly reduced (*p* > 0.001) IL-6 level in HC (Figure 4(B)). LPS after 24 h, showed significant (*p* > 0.001) increase in hippocampal TNF-α level, another proinflammatory cytokine, in mice. Pre-treatment with imipramine and ZAHE at both the dosages significantly (*p* > 0.001) decreased TNF-α level in HC (Figure 4(C)). Compared with the vehicle-treated group, administration of LPS showed a significant decrease in the anti-inflammatory cytokine, IL-10 levels (*p* > 0.001). However, administration of ZAHE (100 and 200 mg/kg) significantly increased the IL-10 levels, as compared to LPS-treated group (*p* > 0.01) (Figure 4(D)). In addition, imipramine pretreatment also improved hippocampal IL-10 level significantly (*p* > 0.001) (Figure 4(D)). Figure 4(E) depicted that after 24 h of LPS administration, the level of another proinflammatory cytokine IL-2 was significantly increased, whereas, imipramine and ZAHE (100 and 200 mg/kg) significantly reduced the level of IL-2.

LPS significantly reduced the hippocampal BDNF level (*p* > 0.01) when compared to vehicle-treated group. ZAHE (200 mg/kg) significantly (*p* > 0.01) elevated BDNF level in the HC of mice as compared to that of LPS treated group. Similarly, imipramine pretreatment prevented the reduction of BDNF level in HC as compared to that of LPS treated group (Figure 4(F)).

#### Effect of ZAHE on brain catecholamines level

**Figure 5.**
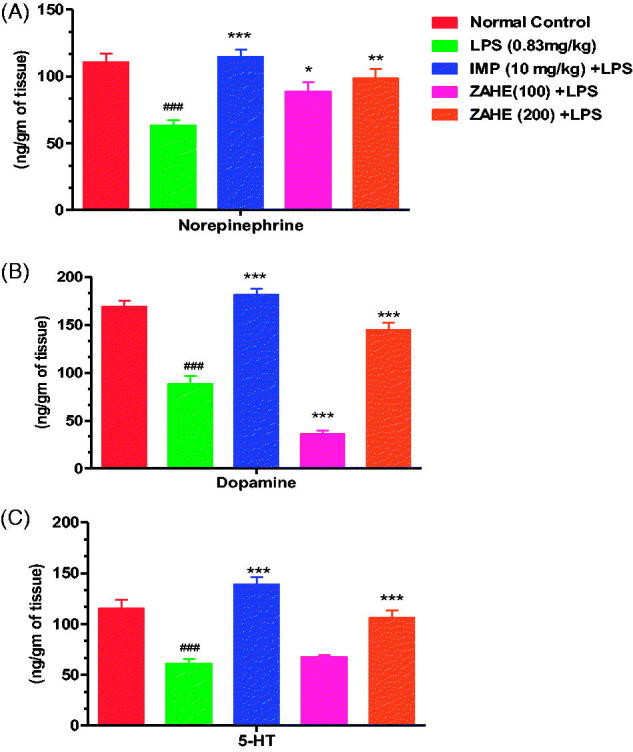
Effect of ZAHE pretreatment in LPS challenged animals on (A) NE, (B) DA and (C) 5-HT in mice HC. Values represent the mean ± SEM. (*N* = 6 animals/group). ###*p* < 0.001, ##*p* < 0.01, #*p* < 0.05 compared with normal control. *p < 0.05; **p < 0.01; ***p < 0.001 compared with LPS challenged group.

The effect of ZAHE on NE, DA and 5-HT levels in the HC in LPS mice is shown in Figure 5(A–C). LPS significantly reduced NE, DA and 5-HT concentrations in the HC compared to vehicle-treated group. Pretreatment with ZAHE (100 or 200 mg/kg) antagonized LPS-induced decline in NE, DA and 5-HT concentrations in the HC. Likewise, imipramine also reverted LPS-induced decrease in catecholamine and 5-HT levels in the HC.

## Discussion

Depressive disorder is a consequence of complex interactions between biological, social and psychological factors. Previous studies revealed that various diseases such as cancer, cardiovascular disorders, diabetes, multiple sclerosis, stroke, Parkinson’s and Huntington diseases may provoke depression (Gustafson et al. 1995; Musselman et al. 1998; McDonald et al. 2003; Patten et al. 2003; Codori et al. 2004; Massie 2004; Ali et al. 2006). The depression-like behaviour of mice in LPS-treated group was studied by behavioural parameters such as FST, TST and SPT. The latency to float and the duration of immobility in the FST are the major parameters associated with depressive-like behaviour in mice. It is a reliable behavioural despair test and has been extensively used to screen new antidepressant drugs (Xue et al. 2015). FST and TST are the most commonly used animal models for the evaluation of antidepressant activity due to their high-predictive validity. SPT is a critical symptom of depression, to represent the behavioural change (Xue et al. 2015). In the present study, our results confirmed that the administration of ZAHE significantly reduced the immobility time and reversed the reduced sucrose consumption.

Imipramine is a tricyclic antidepressant; it blocks the reuptake of neurotransmitters serotonin and NE almost equally. Our results suggested that imipramine-treated group showed increased body weight more significantly than those treated with ZAHE. Furthermore, imipramine ameliorated LPS-induced depression-like behaviour in rat decreased anhedonia, anorexia, weight loss, reduced social, locomotor and exploratory behaviour (Yirmiya 1996; Yirmiya et al. 2001). This was also noticeable in our study.

Proinflammatory cytokines like IL-1β, IL-6, TNF-α and IFN-γ are markers involved in the pathophysiology of depression. These cytokines trigger neuroinflammation and microglial activation resulting pathophysiological changes in depression (Leonard and Maes 2012). In the present study, we have shown that LPS administration elicits increase in the pro-inflammatory cytokines (IL-1β, IL-6, IL-2 and TNF-α) level and reduces the hippocampal IL-10 level. BDNF, a marker for depression, was also reduced in LPS-induced depressed mice. Here our results clearly showed that ZAHE pretreatment antagonized pro-inflammatory responses and elevated BDNF, diminishing the subsequent depressive-like behaviour induced by LPS administration. Moreover, we demonstrated that ZAHE pretreatment significantly improved sucrose intake (anhedonic response) in mice. LPS-induced elevated cytokines are mainly responsible for the behavioural alterations including anhedonic behaviour through augmentation of serotonin transporters (van Heesch et al. 2013). In addition, the anti-inflammatory cytokine, IL-10 ameliorates LPS-induced sickness behaviour and depressive symptoms in transgenic mice (Harvey et al. 2006; Mesquita et al. 2008; Roque et al. 2009). Thus, in our study, reduction of pro-inflammatory cytokines and increase in anti-inflammatory cytokine by ZAHE pretreatment may explain the abandonment of LPS-induced anhedonic behaviour, as one of the contributory factor. Although contradictory reports on the linkage between neuroinflammation and depression by different workers are available.

Oxidative and nitrosative stress pathways play an important role in the pathophysiology of depression (Maes et al. 2011). Pro-inflammatory cytokines increase production of ROS and NO via stimulating the expression of iNOS gene by inducing NF-kB through pro-inflammatory cytokines (Madrigal et al. 2002). Ultimately, NO together with ROS causes oxido-nitrosative stress which lead to neurotoxic effects by reducing monoamines, BDNF in the brain (Vaváková et al. 2015). LPO, high nitrite level, and lower level of antioxidant enzymes are the predisposing factors which are involved in the neuroprogression during depression (Maes et al. 2011). Our results showed that the HC of the mice was affected by LPS-induced oxido-nitrosative stress. MDA level, a marker of LPO, was significantly increased in the HC of LPS-treated mice as compared to that of vehicle-treated group. Reduced level of GSH content indicates the lower antioxidant status of the LPS-treated mice. Nitrite level, an indirect measure of NO content, is found to be increased in HC of LPS challenged mice. ZAHE significantly inhibited MDA and nitrite level whereas, GSH level was significantly improved suggesting that ZAHE attenuates oxido-nitrosative stress in HC of LPS-treated mice. Many earlier studies suggested prominent role of NO in the pathophysiology of depression. Various NOs inhibitors exhibit antidepressant activity via inhibition of NO synthase enzyme in the HC or by reducing the level of cyclic guanosine monophosphate (Kaster et al. 2005; Joca and Guimarães 2006).

In the present study, we have observed that ZAHE administration effectively elevated 5-HT and NE levels in LPS challenged mice. The patterns of changes are similar with standard antidepressant drug imipramine used in this study. Since imipramine is a tricyclic antidepressant involved in reuptake of NE and 5-HT, our compound may also exert its antidepressant effect by similar mechanism like imipramine. Finally, it may be hypothesized that the antidepressant activity of ZAHE could be mediated through multiple mechanisms, i.e., alteration of the monoaminergic responses, antioxidant property and reducing neuroinflammation.

## Conclusions

Our results indicated that the antidepressant-like action of ZAHE was mediated partly via the alteration of monoaminergic response and repression of pro-inflammatory cytokines and oxidative stress in the HC. Further studies are ongoing to investigate the molecular mechanisms of the interactions between monoaminergic neurotransmitter metabolism and inflammation.
